# Probiotic *Escherichia coli* Nissle 1917-derived outer membrane vesicles modulate the intestinal microbiome and host gut-liver metabolome in obese and diabetic mice

**DOI:** 10.3389/fmicb.2023.1219763

**Published:** 2023-08-15

**Authors:** Jun Shi, DongXue Ma, ShanHu Gao, Fei Long, Xin Wang, XingYu Pu, Richard D. Cannon, Ting-Li Han

**Affiliations:** ^1^State Key Laboratory of Ultrasound in Medicine and Engineering, College of Biomedical Engineering, Chongqing Medical University, Chongqing, China; ^2^Chongqing Key Laboratory of Biomedical Engineering, Chongqing Medical University, Chongqing, China; ^3^Department of Oral Sciences, Faculty of Dentistry, Sir John Walsh Research Institute, University of Otago, Dunedin, New Zealand; ^4^Department of Obstetrics and Gynaecology, The Second Affiliated Hospital of Chongqing Medical University, Chongqing, China

**Keywords:** probiotics, *Escherichia coli* Nissle 1917, outer membrane vesicles, diabetes, obesity, gut microbiota, metabolomics

## Abstract

**Introduction:**

Obesity and diabetes are common chronic metabolic disorders which can cause an imbalance of the intestinal flora and gut-liver metabolism. Several studies have shown that probiotics, including *Escherichia coli* Nissle 1917 (EcN), promote microbial balance and metabolic health. However, there are no studies on how EcN outer membrane vesicles (EcN-OMVs) influence the intestinal microflora and affect the metabolic disorders of obesity and diabetes.

**Methods:**

In this study, we evaluated the effects of EcN-OMVs on high-fat diet (HFD)-induced obesity and HFD + streptozotocin (STZ)-induced diabetes.

**Results:**

EcN-OMVs could reduce body weight, decrease blood glucose, and increase plasma insulin in obese mice. Similarly, EcN-OMVs treatment could modify the ratio of *Firmicutes*/*Bacteroidetes* in the gut, elevate intestinal short-chain fatty acid (SCFA)-producing flora, and influence the SCFA content of the intestine. Furthermore, the intestinal metabolites ornithine and fumaric acid, hepatic ω-6 unsaturated fatty acids, and SCFAs were significantly increased after administering EcN-OMVs.

**Discussion:**

Overall, this study showed that EcN-OMVs might act as post-biotic agents that could modulate gut-liver metabolism and ameliorate the pathophysiology of obesity and diabetes.

## 1. Introduction

The incidences of obesity and diabetes are increasing dramatically, the rates are assuming pandemic proportions. It has been estimated that approximately 57.8% of the world’s adult population will be obese or overweight by 2030 (Kelly et al., 2008; Meldrum et al., 2017). The global incidence of diabetes has been predicted to increase from 451 million (age 18–99 years) people in 2017 to 693 million cases in 2045 (Cho et al., 2018; Khan et al., 2020). Obesity and diabetes can also cause a variety of associated health problems, such as elevated blood glucose, cardiovascular disease, osteoarthritis, kidney disease, fatty liver, sleep apnoea and intestinal microbial flora disorder (Bluher, 2019; Harding et al., 2019). Therefore, there is an urgent need to discover new prevention and treatment strategies.

Interestingly, a previous study found that the gut microbiota influences nutrient acquisition and energy regulation in the host, as well as the development of obesity, insulin resistance, and diabetes (Durack and Lynch, 2019). Hence, microbiota manipulation through diet changes has been postulated as a promising therapeutic approach. It is increasingly recognized that probiotics can contribute to preventing obesity and alleviating diabetes by manipulating the intestinal microbiota composition and the production of various metabolites (Sommer and Backhed, 2013). Many probiotics have been promoted as pharmaceutical products or dietary supplements to improve obesity, diabetes, and intestinal dysfunction (Sanders et al., 2019; Vallianou et al., 2020). *Escherichia coli* Nissle 1917 (EcN) is a probiotic strain isolated by Alfred Nissle from the stools of a soldier who was not infected during an outbreak of shigellosis, and is used in several probiotic products. EcN is not pathogenic because its lacks virulence factor genes in its genome. It has been reported that EcN colonizes the human gut readily and promotes intestinal homeostasis and microflora balance (Hancock et al., 2010; Scaldaferri et al., 2016; Geervliet et al., 2022).

There is a large amount of evidence indicating that probiotic-mediated effects are mostly achieved indirectly. Studies have shown that almost all Gram-negative, and some Gram-positive, bacteria release nanometer-sized membrane vesicles called extracellular vesicles (EVs) (Ahmadi Badi et al., 2017; Woith et al., 2019). EVs produced by Gram-negative bacteria are derived from the outer membrane and are thus termed as outer membrane vesicles (OMVs). These spherical bilayered phospholipid structures could act as vehicles to mediate gut microbiota-host communication (Kim et al., 2015, 2017). Purified OMVs contain a diverse array of bioactive molecules, such as lipids, proteins, lipopolysaccharides (LPS), phospholipids, and nucleic acids, that could reach host cells, modulate essential biological functions and influence host health (Furuyama and Sircili, 2021). EVs derived from specific bacteria can induce different physiological responses. For example, Raftar and colleagues have shown that *Akkermansia muciniphila* EVs can act as a mucosal delivery vector to reduce the deleterious consequences of obesity in mice. *A. muciniphila* EV treatment caused a significantly greater loss in body weight and fat in HFD mice than treatment with the bacterium itself. Similarly, the same authors found that both *A. muciniphila* and its EVs improved blood glucose and lipid levels in obese mice, and were significantly correlated with intestinal homeostasis (Raftar et al., 2021, 2022). In addition, Qu et al. (2022) have suggested that probiotic-derived vesicles could repair tissue damage associated with the infection by upregulating the levels of anti-inflammatory factors, downregulating pro-inflammatory factors, and regulating cellular biological behaviors. Furthermore, Hu et al. (2020) demonstrated that EcN-OMVs could modulate the functions of host immune cells by stimulating RAW264.7 macrophage proliferation, phagocytic functions, and immune-related enzymatic activities. Another study has shown that EcN-OMVs could ameliorate dextran sodium sulfate-induced mucosal injury and inflammation in the gut, and maintain the intestinal barrier function (Fabrega et al., 2017). However, there is no study investigating the EcN-OMV regulation of metabolic disorder the effect of EcN-OMVs on microbial flora composition in obese and diabetic mice.

In this study, we aimed to investigate the effects of EcN-OMVs on metabolic dysfunctions in mice (obesity and diabetes) by using microbiome and metabolomic approaches. We also studied the mechanism of how EcN-OMVs act as a probiotic-derived therapeutic approach to alleviate obesity and diabetes.

## 2. Materials and methods

### 2.1. Bacterial culture

The probiotic *E. coli* strain Nissle 1917 (EcN) was purchased from Biobw (Beijing, China). The bacteria were grown at 37°C in Luria-Bertani (2 g/L, LB) broth with continuous shaking at 180 rpm until the culture reached exponential phase.

### 2.2. OMV isolation

Outer membrane vesicles (OMVs) were isolated from the EcN culture supernatant as described previously (Fabrega et al., 2016). In brief, the *E. coli* cells were removed from the culture by centrifugation at 5,000×*g* for 30 min at 4°C. Then the supernatant was centrifuged at 10,000×*g* for 30 min at 4°C. The collected supernatant was sequentially filtered through 0.45 μm and 0.22 μm pore size polyethersulfone membranes (Sorfabio, Beijing, China) to remove large particles such as bacterial residues and cellular debris. The filtered supernatant was then ultracentrifuged at 150,000×*g* for 3 h at 4°C to isolate EcN-OMVs. The EcN-OMV pellet was resuspended in sterile phosphate buffered saline (PBS; pH = 7.4). The EcN-OMVs were stored at −80°C for later use.

### 2.3. Transmission electron microscopy

Isolated OMVs were imaged by transmission electron microscopy (TEM) after negative staining as described by Aguilera et al. (2014). A drop of OMV suspension was placed on Formvar/carbon coated-grids that were previously activated by UV light, for 2 min. Grids were washed with deionized water, stained with 2% uranyl acetate for 1 min, air dried, and evaluated by TEM (Jeol, JEM 1010, Japan).

### 2.4. NanoSight tracking analysis

A NanoSight NS300 (United Kingdom) optical nanoparticle Brownian motion imager was used to measure the size of OMVs. The number of particles and their movement was recorded for 5 × 60 s (camera level = 11). Particle sizes were quantified by nanotracking analysis (detection threshold = 5) using the NS500 software.

### 2.5. Immunoblotting

The OMVs were mixed with SDS–PAGE sample buffer, heated for 5 min at 95°C, and the proteins were separated using 12% SDS-PAGE gels (Beyotime, China) followed by transferring onto 0.45 μm PVDF membranes (Millipore, United States). The membranes were blocked with PBS-0.05% Tween-20 and 5% skimmed milk and incubated with primary antibodies (OMPA and OMPC, Abcam, United States) in 8% BSA solution overnight at 4°C. After washing, the blots were incubated with appropriate secondary antibodies (Beyotime, China) for 1 h at room temperature. Lastly, the blots were visualized using the Genegenome XRQ Chemiluminescence system (Syngen, United Kingdom).

### 2.6. Establishment of obesity and diabetes mouse models

**Figure 1:**
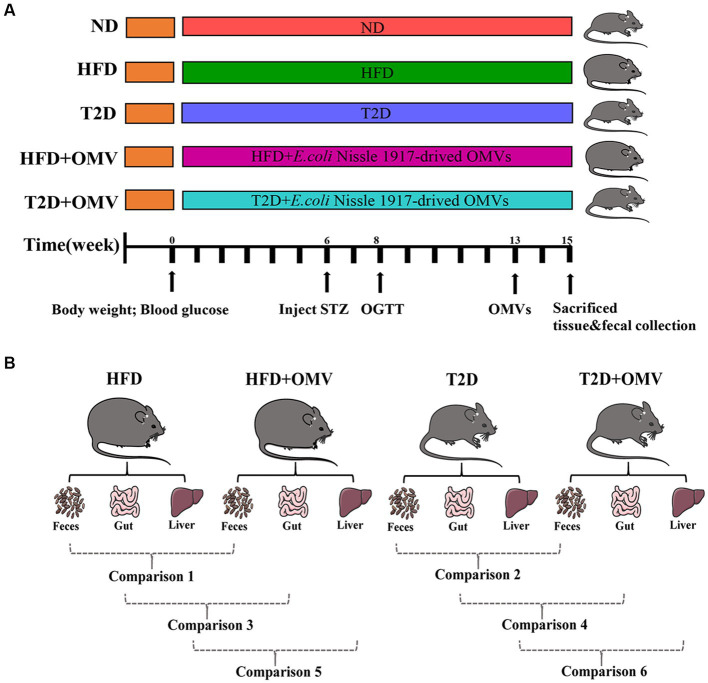
**(A)** Schematic diagram of study design. The important events are indicated on the timeline. There are five experimental groups abbreviated as follows; ND, normal diet; HFD, high-fat diet; T2D (type 2 diabetes), high-fat diet + STZ; HFD + OMV, high-fat diet + OMVs; T2D + OMV, high-fat diet + STZ + OMVs. **(B)** Data comparisons in this study. Comparison 1 compares the fecal metabolites between the HFD + OMV group and the HFD group. Comparison 2 compares the fecal metabolites between the T2D + OMV group and the T2D group. Comparison 3 compares the gut metabolites between the HFD + OMV group and the HFD group. Comparison 4 compares the gut metabolites between the T2D + OMV group and the T2D group. Comparison 5 compares the liver metabolites between the HFD + OMV group and the HFD group. Comparison 6 compares the liver metabolites between the T2D + OMV group and the T2D group.

Mice were raised in accordance with the “Guide for the care and the Use of Laboratory Animals” as promulgated by the Institutional Animal Care and Use Committee (IACUC) of the Chongqing Medical University. The methodology was approved by the Ethics Committee of the Chongqing Medical University. Male C57BL/6 J mice (8-weeks-old; approximately 18 g) were obtained from the Experimental Animal Center of Chongqing Medical University. Firstly, all mice were collectively reared adaptively for 2 weeks, and then randomly assigned into five groups: normal diet group (ND), obesity group (HFD), diabetes group (T2D), obesity with EcN-OMVs treatment group (HFD + OMV), and diabetes with EcN-OMVs treatment group (T2D + OMV) (Figure 1A). Moreover, the success rates of the induction mice model in each group were 100, 75, 75, 100, and 100% for ND, HFD, T2D, HFD + OMV, and T2D + OMV, respectively. The ND group was fed with the normal mouse growth diet (20.6% kcal protein, 67.4% kcal carbohydrate, and 12% kcal fat with 3.6 kcal/g), and the other four groups (HFD, T2D, HFD + OMV, T2D + OMV) were fed with the high-fat diet (20% kcal protein, 20% kcal carbohydrate, and 60% kcal fat with 5.24 kcal/g) for a total of 15 weeks. Animal body weight and blood glucose were measured weekly. The diabetic mice (T2D and T2D + OMV) were given 40 mg/kg of streptozotocin (STZ) by intraperitoneal injection for 3 days in the sixth week. The obesity mice (HFD and HFD + OMV) did not receive any other intervention and were maintained on a high-fat diet. All groups were subjected to an oral glucose tolerance test (OGTT) in the eighth week. The experimental design is shown in Figure 1A.

### 2.7. Gastric gavage with OMVs

After the mouse models were stably established in the 15th week, the treatment groups were given 500 μL of OMVs (17.5 μg protein/500 μL) by gavage every day for 2 weeks, and the same amount of PBS was given to the control group. The 2-weeks OMV treatment was adopted based on previous studies (Fabrega et al., 2017; Chelakkot et al., 2018; Du et al., 2021). The concentration of OMVs was measured using a BCA Protein Assay Kit (Beyotime, Shanghai, China).

### 2.8. 16S rRNA gene sequencing of gut microbiota

The gut microbiota was analysed by 16S rRNA gene sequencing of the fecal samples of all groups of mice. This was performed by the Chongqing Puroton Institute of Genetic Medicine Co., Ltd.; the main sequencing process was as follows: (1) DNA extraction and PCR amplification: Fecal microbial DNA was extracted using a DNA extraction kit (Qiagen, United States). Universal primers for 16S rRNA (338F and 806R) containing inducers and sequencing adaptors were employed to amplify the V3-V4 gene regions. The sequencing read length for MiSeq was 2 × 300 bp. PCR amplification was performed using a polymerase mix (New England Biolabs). (2) Purification and recovery of amplification products: DNA amplicons were analyzed using 2% agarose gel electrophoresis, and the GeneJET gel recovery kit (Thermo Scientific) was used to recover the amplification products. (3) Quantification of amplification products: the PCR products were quantified using the Quant-iT PicoGreen dsDNA Assay Kit. (4) Preparation of sequencing library: the TruSeq NanoDNA LT Library Prep Kit (Illumina) was used to prepare the sequencing library, and the sequencing libraries were quantified using Qubit. (5) High-throughput sequencing: After performing sequencing library quality control, MiSeq was used to obtain the 16S rRNA sequences. Sequences were binned into OTUs and taxonomy was assigned by QIIME software using Greengenes database (version gg_13_8). All the sequencing data are available in the NCBI Sequence Read Archive database (Citation accession: PRJNA971528).

### 2.9. Glucose measurement and mouse sample collection

All blood samples were collected from the tail vein, and glucose was measured by the glucose oxidase method using a hand-held OneTouch Ultra glucometer (Sinocare, Beijing, China). To perform the oral glucose tolerance test (OGTT), basal blood glucose levels were first measured after 15 h overnight fasting. A 40% (wt/vol) of glucose solution was then intragastrically administrated at 2 g/kg, and blood glucose levels were measured at 15, 30, 60 and 120 min after the glucose loading. If the glucose level was greater than 599.4 mg/dL, the value of 599.4 mg/dL was recorded. The fecal samples were collected weekly throughout the whole feeding period. Two weeks after the completion of the vesicle gavage intervention, the mice were sacrificed by neck dislocation and samples of liver, gut, and plasma were obtained. The samples for metabolic analysis were frozen at −80°C.

### 2.10. Histological analyses

Liver and gut biopsies were fixed in 4% formaldehyde/phosphate buffer for 24 h at 4°C, then dehydrated and embedded in paraffin. Tissue sections (5 μm) were stained with hematoxylin and eosin. Light microscopy with Dino-lite digital lens and Dino Capture 2 software (AnMo Electronics Corp., Taiwan) was used for histopathological analysis.

### 2.11. Short-chain fatty acid (SCFA) quantification by solid phase micro-extraction (SPME) GC–MS

#### 2.11.1. Preparation of mouse fecal samples

A salt solution containing 1.26 g/mL of (NH_4_)_2_SO_4_/NaH_2_PO_4_ in a 3.7:1 ratio and 0.5 mM of internal standard D4-acetic acid was prepared to improve SCFA extraction efficiency and reproducibility. Fecal samples (20 mg) were transferred to a 2 mL screw cap tube with 400 µL of the salt solution and tungsten carbide beads (3 mm diameter). The fecal mixture was homogenized using a TissueLyser II (QIAGEN, United States) and transferred into a 20 mL glass vial with 1.6 mL of the salt solution. The glass vials were placed in the autosampler (PAL RTC 120, Agilent, United States) of the SPME-GC–MS machine until analysis.

#### 2.11.2. SPME-GC–MS analysis

The SPME method used to analyze SCFA was adapted from Nylund et al. (2020). SCFAs were extracted with an SPME fiber on an Agilent Auto-sampler (PAL RTC 120), separated by a 5977A MSD gas chromatograph (Agilent) using a DB-FFAP (30 m × 250 μm id × 0.25 μm, Agilent), and analyzed with a 7890B mass spectrometer (Agilent).

SPME conditions were as follows: DVB/CAR/PDMS fiber (Agilent), agitation temperature 35°C with an extraction time of 30 min. Temperature and desorption time had been preliminarily evaluated and set at 260°C and 5 min, respectively. The fiber post-injection condition was performed at 270°C for 10 min.

The GC conditions were as follows. The volatile compounds were injected into an inlet with a splitless mode at 260°C with 1 mL/min flow rate of helium gas. The GC-oven program was initially held at 35°C for 4 min. Then the temperature was elevated from 35°C to 130°C at a rate of 70°C/min. Then the temperature was ramped at 5°C/min until it reached 155°C. Lastly, the temperature was raised to 240°C at a rate 120°C/min and held for 4 min; the total run time was 15.06 min.

The MS conditions were as follows. The temperatures of auxiliary, MS quadrupole, and MS source were 250°C, 230°C, and 150°C, respectively. The mass range was detected from 30 μm to 550 μm. Scan speed was set to 1.563 μ/s, and the solvent delay was applied until 5.0 min.

### 2.12. Metabolomic profile by methyl chloroformate derivatization (MCF) based GC–MS analysis

#### 2.12.1. Preparation of fecal, liver, and gut samples

Briefly, fecal and tissue samples were thawed on ice at 4°C and portions (30 mg) transferred from cryotubes to 1.5 mL microcentrifuge tubes. Sodium hydroxide (1 M) and methanol mixture (1:1 v/v; NaOH/MeOH 0.4 mL), two tungsten carbide beads (3 mm diameter), and 10 μL of D4-alanine (10 mM) were added to each sample followed by 30 s vortex mixing. The samples were homogenized using a TissueLyser II (QIAGEN, United States) at 30 Hz for 1 min. Then the supernatant was isolated by centrifugation at 12,000 rpm (4°C) for 15 min and stored at 4°C prior to derivitization.

#### 2.12.2. OMV sample preparation

Isolated OMVs (500 μL) were concentrated by freeze-drying using a SpeedVac (Labconco, United States) for 4 h at 0.8 HPa. The dried pellets were resuspended in 0.4 mL of NaOH/MeOH and 10 μL of D4-alanine (10 mM) was added followed by 30 s vortex mixing. Then the supernatant was isolated by centrifugation at 12,000 rpm (4°C) for 15 min and stored at 4°C prior to derivitization.

#### 2.12.3. MCF derivitization

To stored supernatants 34 μL of pyridine and 20 μL MCF was added, followed by 30 s of vortex mixing, then another 20 μL of MCF was added followed by 30 s of vortexing. Then, 200 μL of chloroform and 400 μL of sodium bicarbonate (50 mM) were added and the solutions vortexed for 10 s. Subsequently, the aqueous layer was separated from the chloroform layer by centrifugation at 2,000 rpm for 10 min. After centrifugation, the aqueous layer was removed and the remaining chloroform extract was dehydrated by the addition of sodium sulphate (~0.3 g), then transferred to an amber glass GC–MS vial. Negative controls were produced by subjecting an empty microcentrifuge tube to the same processing as the samples.

#### 2.12.4. GC–MS analysis

The GC–MS instrument parameters were set according to Gao et al. (2022). Specifically, the derivatized metabolites were analyzed using an Agilent Intuvo9000 coupled to a MSD5977B with 70 eV of electron impact ionization. The gas capillary column was a BD-1701 (30 m × 250 μm id × 0.25 μm, Agilent). The derivatized samples were injected into a pulsed splitless mode inlet at 290°C with 1 mL/min flow rate of helium gas. The GC-oven program was initially held at 45°C for 2 min. Then the temperature was elevated from 45°C to 180°C at a rate of 9°C/min and held for 5 min. Then the temperature was ramped at a rate of 40°C/min until it reached 220°C and held for 5 min. Then the temperature was raised to 240°C at a rate of 40°C/min and held there for 11.5 min. Lastly, the temperature was raised to 280°C at a rate 80°C/min. The temperatures of the guard chip, auxiliary, MS quadrupole, and MS source were 280°C, 250°C, 230°C, and 150°C, respectively. The mass range was detected from 30 μm to 550 μm. The scan speed was set to 1.563 μ/s and the solvent delay was applied until 5.5 min.

### 2.13. Data extraction and normalization

Automated Mass Spectral Deconvolution & Identification System software was used for metabolite deconvolution and identification. The metabolites were identified by comparing the MS fragmentation patterns (relative intensity of mass spectra to the most abundant ion) and GC retention time within 0.5 min bins to our in-house MS library built using chemical standards. The MassOmics R-based program was implemented to extract the relative concentration of the metabolites using the peak height of the most abundant reference ion mass. To facilitate quantitative robustness along with minimizing instrumental and human variability, the relative concentrations of the identified compounds were normalized with internal standards (D4-alanine) and then adjusted for either protein concentration or weight of samples for bacterial OMVs or mice samples (fecal, liver, gut).

### 2.14. Statistical analysis

With regard to the microbiome results, alpha diversity (Simpson, Shannon, and evenness indices), and rarefaction curves were analyzed and plotted using the website https://www.bioincloud.tech/. Phylogenetic beta diversity measures, weighted and unweighted UniFrac distance matrices were calculated using QIIME and visualized with principal coordinate analysis (PCoA). Linear discriminant analysis effect size (LEfSe) analysis was used to identify taxa significantly enriched in the treatment groups. The linear discriminant analysis (LDA) score was computed for taxa differentially abundant between the control group and the treatment group. A taxon at *p* < 0.05 (Kruskal–Wallis test) and log_10_[LDA] ≥2.0 (or ≤ −2.0) were considered significant. The Analysis of Composition of Microbiomes (ANCOM) was performed on QIIME and applied to identify differentially abundant features across the five groups (ND, HFD, T2D, HFD + OMV, and T2D + OMV).

Metabolomic data are presented as the mean ± SEM. The comparisons between two groups were conducted using an unpaired Student’s t-test. Comparisons between five groups (ND, HFD, T2D, HFD + OMV, T2D + OMV) were determined using two-way ANOVA followed by Tukey post-hoc analysis. The false discovery rates (FDRs) were calculated using the q-value function in the R program for accounting for multiple comparisons. The important variables in the partial least squares discriminant analysis (PLS-DA) projection were determined using the ropls R-package. *p* < 0.05 with corresponding q-value (FDR) < 0.3 were considered statistically significant. Metabolic pathway activity was predicted based on the Pathway Activity Profiling (PAPi) R-algorithm. Correlations between gut microbiota and metabolites were analyzed by Pearson’s correlation using R. Bar graphs and line plots were illustrated using GraphPad Software Prism 9 (GraphPad Software, San Diego, United States). A graphical representation of the significant metabolites was displayed in heat maps using the ggplot2 and complexheatmap R packages.

## 3. Results

### 3.1. Identification of EcN-OMVs

**Figure 2:**
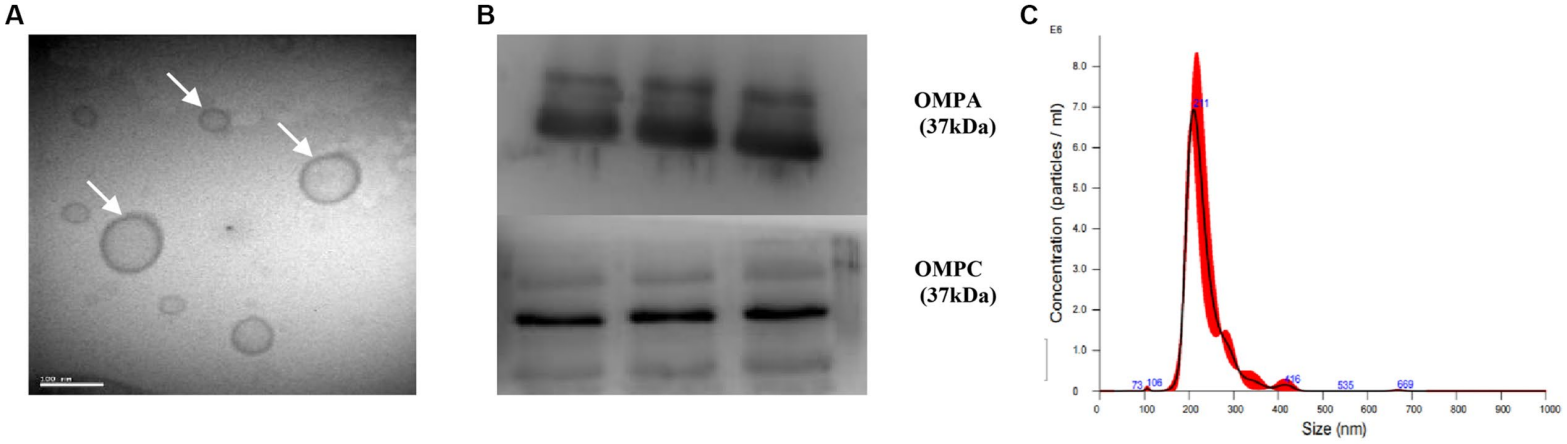
Characterization of EcN-OMVs. **(A)** Transmission electron micrograph displaying the morphology of OMVs. **(B)** Western blotting validating OMVs through expression of OMPA and OMPC. **(C)** The size distribution of isolated OMVs measured by nanoparticle tracking analysis.

The morphology of the OMVs and their size distribution were characterized by transmission electron microscopy (Figure 2A) and nanoparticle tracking analysis (Figure 2C), respectively. We observed that the OMVs were spherical particles with a size range of 20–300 nm, with the most abundant particle size being 211 nm. Furthermore, western blot analysis verified that the isolated OMVs shared several protein markers with probiotic EcN including outer membrane protein A (OMPA) and outer membrane protein C (OMPC) (Figure 2B). These results indicated that OMVs were successfully isolated from the EcN strain.

### 3.2. Body weight, blood glucose and oral glucose tolerance test (OGTT) of obese and T2D mice prior to OMV intervention

**Figure 3:**
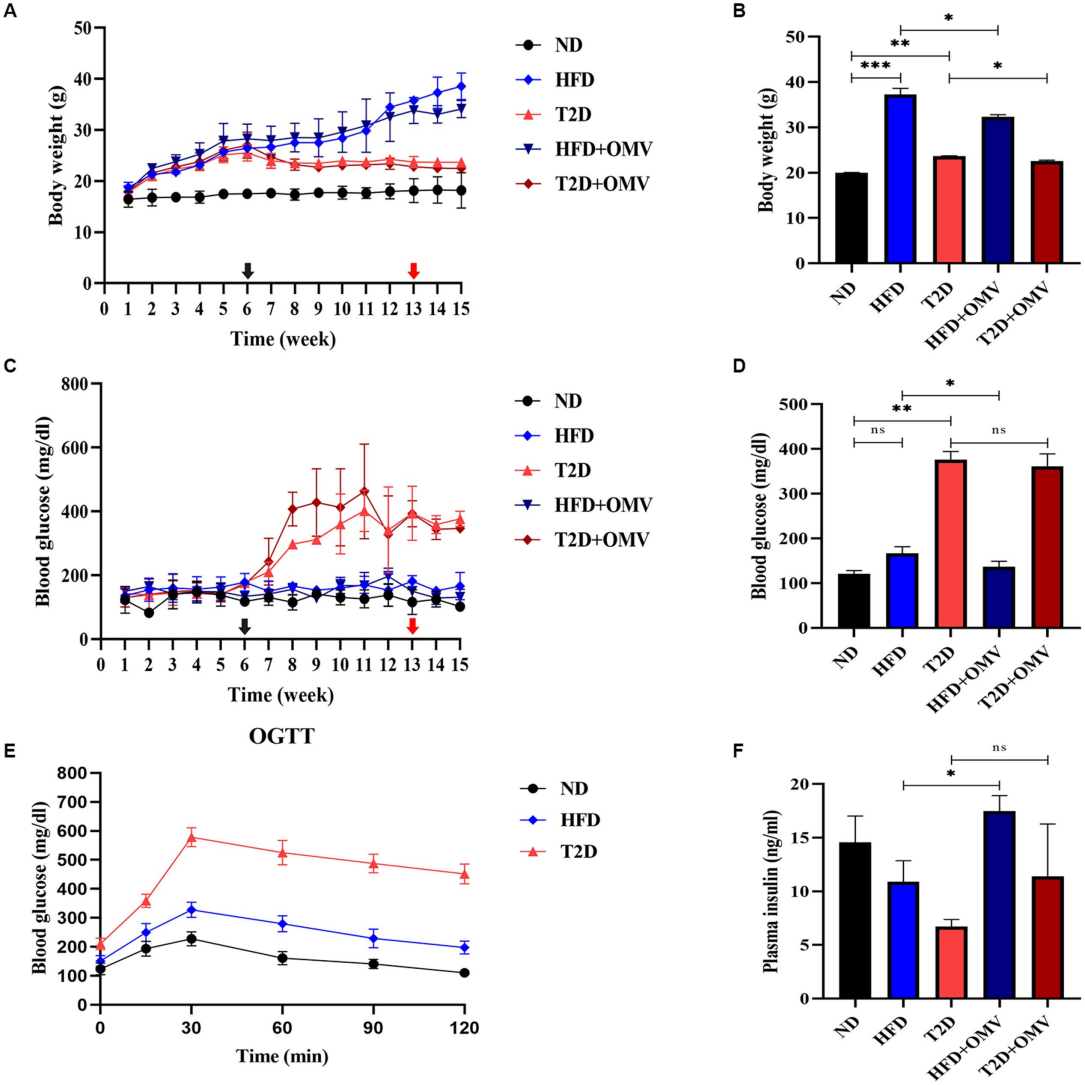
The effects of OMVs on body weight **(A,B)**, blood glucose **(C,D)**, and plasma insulin **(F)** in obese (HFD) and T2D mice. The levels of OGTT in normal, obese, and diabetes mice at 8 weeks **(E)**. The black arrow indicates the time of STZ intraperitoneal injection (6 weeks), while the red arrow indicates the time of EcN-OMV intervention (13 weeks). Bar graphs **(B,D,F)** are outcomes after OMV treatments past 13 weeks. Data are presented as the mean ± SD. Statistical analysis was conducted by one-way or two-way ANOVA followed by the *post hoc* Tukey–Kramer test and Student’s *t*-test, as appropriate. ^*^p ≤ 0.05, ^**^p ≤ 0.01, ^***^p ≤ 0.001.

The diabetic-related characteristics of mice prior to 13 weeks of OMV treatment are depicted in Figure 3. Three was a general increase in weight in non-diabetic mice, and the HFD group showed the greatest weight gain (Figure 3A). All groups of mice had similar blood glucose levels until week 6 when, after STZ injection, the blood glucose concentration increased rapidly in the T2D group and reached more than 288 mg/dL (Figure 3C). OGTT measurements at 8 weeks showed that the highest glucose levels were in the T2D group (Figure 3E). Thus, the T2D group displayed typical diabetes symptoms as upon administration of glucose, the concentration rose rapidly and did not return to physiological levels within 2 h.

### 3.3. Body weight, blood glucose and plasma insulin concentrations of mice after OMV gavage

After gastric gavage feeding with OMVs daily from week 13 to 15, the body weight (Figure 3B) of the OMV treatment groups (HFD + OMV and T2D + OMV) was significantly lower than their corresponding non-OMV groups (HFD and T2D). The blood glucose (Figure 3D) concentrations were significantly lower in the HFD + OMV group but there was no noticeable change for the T2D + OMV group. Similarly, the plasma insulin concentration (Figure 3F) was increased significantly in the HFD + OMV group. Unlike the findings in HFD + OMV mice, the T2D + OMV had no effects on plasma insulin levels. Overall, the OMVs exerted potential preventive effects on obesity and T2D by decreasing body weight, blood glucose, and increasing plasma insulin.

### 3.4. Histology analysis

**Figure 4:**
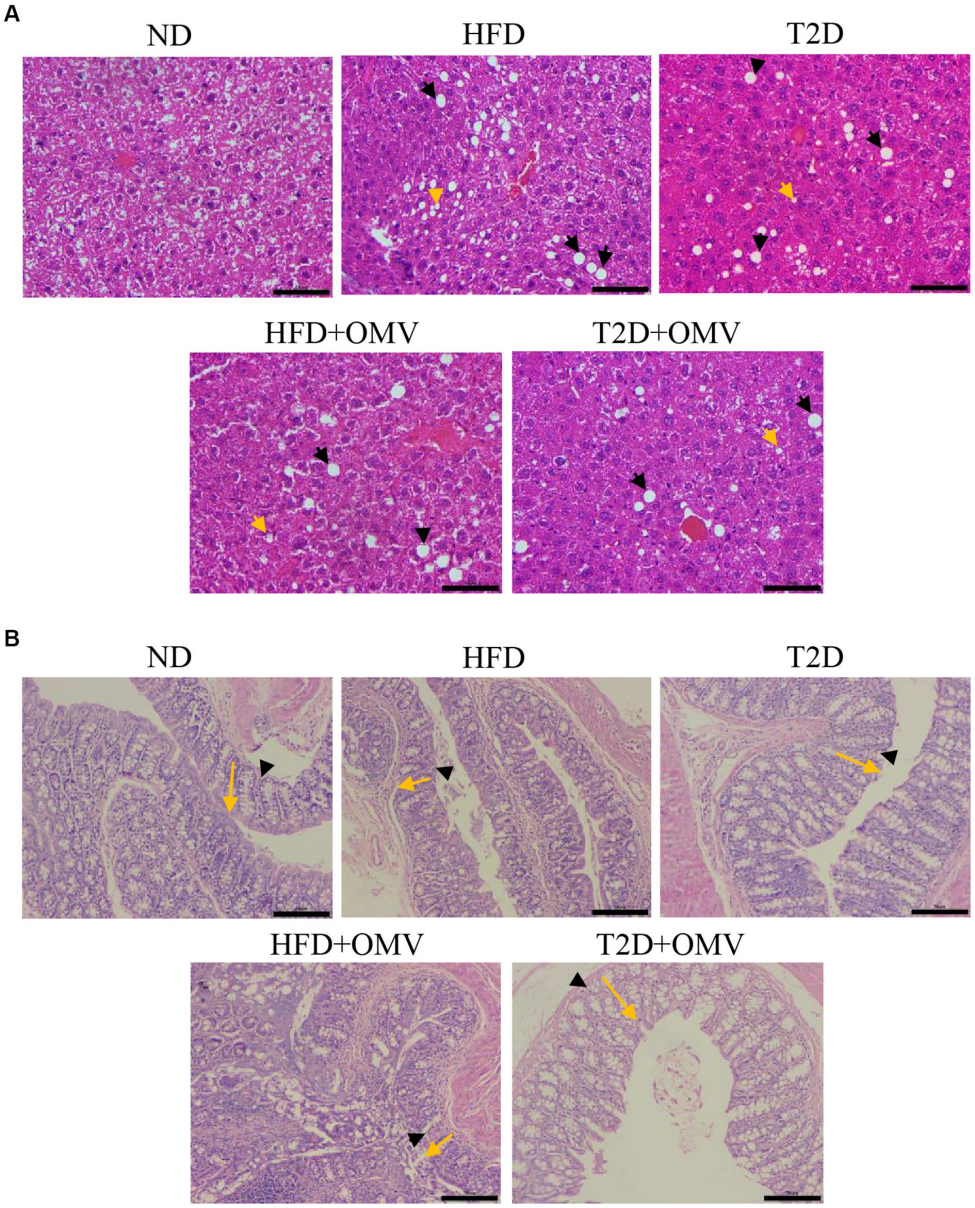
The effects of EcN-OMVs on the histopathology of mouse liver and gut tissues. **(A)** Hematoxylin and Eosin (H&E) staining of liver sections (yellow arrows: lipid droplets in microvesicular form, and black arrows: lipid droplets in macrovesicular form, scale bar is 50 μm). **(B)** H&E staining of gut sections (yellow arrows indicate crypt depth, and black arrowheads indicate mucous thickness, scale bar is 50 μm). ND, normal diet; HFD, high-fat diet; T2D (type 2 diabetes), high-fat diet + STZ; HFD + OMV, high-fat diet + OMVs; T2D + OMV, high-fat diet + STZ + OMVs.

To examine the effects of EcN-OMVs on mice in more detail, we assessed the histopathologies of liver and gut tissues. In the liver (Figure 4A), there was an excessive accumulation of lipid droplets in both macrovesicular and microvesicular forms in the HFD and T2D groups compared with the ND group. The lipid droplets were more prominent in the HFD group than in the T2D group. The OMV treatment in the T2D and HFD mice reduced the number of lipid droplets compared with the non-OMV treatment group. Overall, the histopathological examination of the liver samples indicated that EcN-OMVs could prevent HFD and T2D-induced hepatic steatosis.

Since a high-fat diet altered the morphology and integrity of HFD- and T2D-treated mouse liver, we evaluated gut tissue by H&E staining (Figure 4B). Compared with the ND group, the crypt depth and mucous layer thickness was considerably less in the HFD and T2D groups. Nevertheless, no obvious histopathological changes were observed in the OMV treatment groups.

### 3.5. Overview of differences in the metabolite profiles

**Figure 5:**
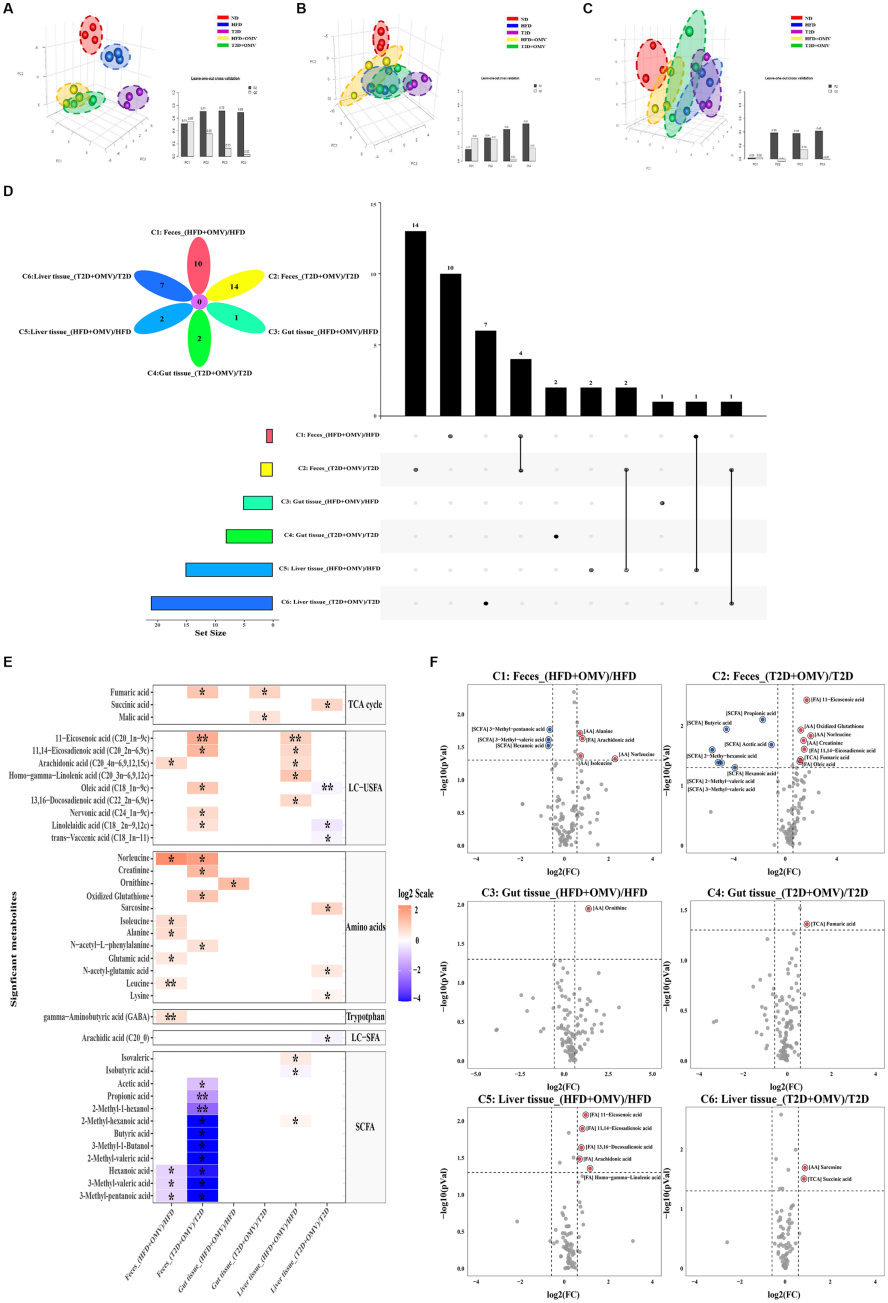
Metabolite analysis. Partial least squares discriminant analysis (PLS-DA) of metabolite patterns in **(A)** feces, **(B)** gut tissue, and **(C)** liver tissue. **(D)** Upset plot and Venn diagram of differential (adjusted *p* < 0.05) metabolite levels. The individual or connected dots represent the various intersections of metabolites that were either unique to, or shared among, comparisons, respectively. **(E)** Heatmap of the metabolites detected in feces, gut tissue, and liver tissue showing the ratio of metabolite levels between groups. Red colors indicate higher metabolite concentrations in OMV treatment groups than the corresponding non-OMV treatment groups, while blue colors indicate lower metabolite concentrations in OMV treatment groups than the corresponding non-OMV treatment groups. The relative concentration of metabolites was plotted using a log_2_ scale. The significant metabolite differences (adjusted *p* < 0.05, FC > 1.5) are labelled with an asterisk*, and more signficant differences (adjusted *p* < 0.001, FC > 1.5) are labelled with a double asterisk**. **(F)** Volcano plot of the differential (*p* < 0.05, FC > 1.5) metabolite levels between groups. Red dots indicated upregulation, while blue dots indicated downregulation in response to the OMV treatment. HFD, high-fat diet; T2D (type 2 diabetes), high-fat diet + STZ; HFD + OMV, high-fat diet + OMVs; T2D + OMV, high-fat diet + STZ + OMVs.

A total of 132 distinct metabolites were identified in feces, gut and liver tissue. Three-dimensional partial least squares discriminant analysis (PLS-DA) was performed. We found that the metabolite profiles in feces (Figure 5A), gut (Figure 5B) and liver (Figure 5C) tissue were all clearly segregated among five groups. The metabolites that were unique or shared among different groups were visualized using an Upset plot (Figure 5D). Interestingly, no common metabolites were found among all six comparisons. Ten, fourteen, one, two, two and seven metabolites were unique for comparisons 1–6 (Figure 1B) respectively (Figure 5D). In T2D comparisons, fourteen and seven metabolites fluctuated significantly in the feces (C2) and liver tissue (C6), respectively, with OMV treatment, while the concentrations of two metabolites significantly increased in the gut tissue with OMV treatment (C4). In the comparison of HFD groups, ten and two metabolites were significantly elevated in the feces (C1) and liver tissue (C5), respectively, with OMV treatment, while only one metabolite’s concentratin was significantly elevated in the gut tissue (C3) with OMV treatment. Thus, OMV treatment has a greater effect on metabolite changes in feces and liver tissue than in intestinal tissue.

### 3.6. Characteristic metabolite analysis

Heat maps and volcano plots were used to show differences in the concentrations of specific metabolites in comparisons 1–6 for feces, gut, and liver tissue (Figures 5E,F). In the fecal metabolome, the concentrations of four metabolites (arachidonic acid, norleucine, alanine, and isoleucine) were elevated, and the concentrations of three SCFAs (3-methyl-pentanoic acid, 3-methyl-valeric acid, and hexanoic acid) were decreased in the OMV group for comparison 1 (FC > 1.5, adjusted *p* < 0.05). For comparison 2, the concentrations of seven metabolites (11-eicosenoic acid, oxidized glutathione, norleucine, creatinine, 11,14-eicosadienoic acid, fumaric acid, and oleic acid) were increased, whilst the concentrations of seven SCFAs (propionic acid, butyric acid, acetic acid, 2-methyl-hexanoic acid, hexanoic acid, 2-methyl-valeric acid, and 3-methyl-valeric acid) were decreased in the OMV group (FC > 1.5, *p* < 0.05). In the gut metabolome, only the concentrations of ornithine and fumaric acid were elevated in the OMV groups for comparison 3 and comparison 4, respectively, (FC > 1.5, *p* < 0.05). In the liver metabolome, the concentrations of unsaturated LCFAs such as 11,14-eicosadienoic acid, 11-eicosenoic acid, 13,16-docosadienoic acid, arachidonic acid, and homo-gamma-linolenic acid were significantly increased in the OMV treatment group for comparison 5. Lastly, the concentrations of sarcosine and succinic acid in the T2D + OMV group were significantly higher than in the T2D group (comparison 6) (FC > 1.5, *p* < 0.05).

### 3.7. Metabolic pathway enrichment analysis

**Figure 6:**
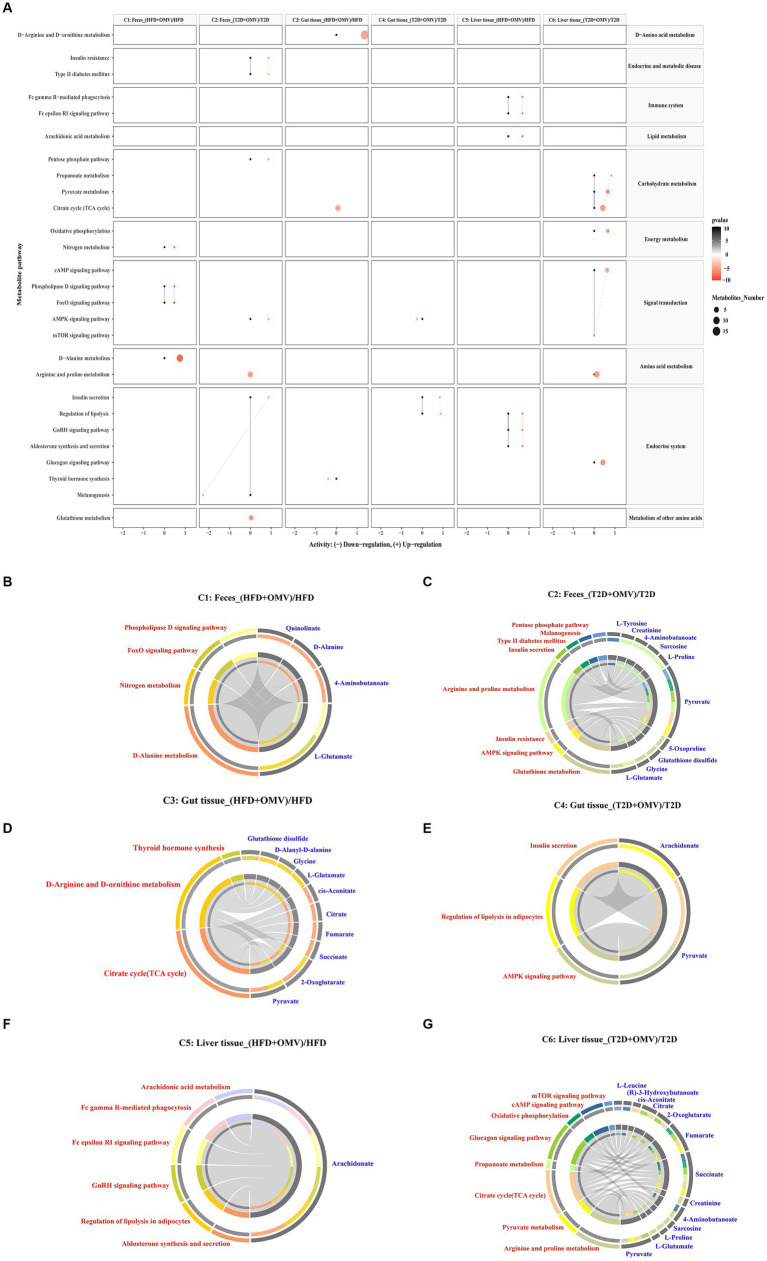
Metabolic pathway enrichment. **(A)** Activities of metabolic pathways in feces, gut tissue, and liver tissue for comparisons 1–6. Black dots represent metabolic activities in feces, gut tissue and liver tissue from the HFD group and the T2D group that were adjusted to 0. Red dots represent metabolic activities in feces, gut tissue, and liver tissue from the HFD + OMV group and the T2D + OMV group. The metabolic activities were visualized using a log_2_ scale. The dot size indicates the number of metabolites involved in the pathway, and the intensity of the red dot color indicates the significance of *p*-value. **(B–G)** Circos plots display the connectivity of significant metabolites and their pathways in feces for comparison 1 **(B)** and comparison 2 **(C)**. The connectivity between significant metabolites and pathways in the gut tissue for comparison 3 is shown in **(D)** and for comparison 4 is shown in **(E)**. Similarly, comparisons 5 **(F)** and comparison 6 **(G)** show the connectivity between significant metabolites and pathways in the liver tissue. In **(B–G)**, the red text signifies the shortlisted metabolic pathways and the blue text represents the significant metabolites.

To further explore the biological role of identified metabolites, we annotated metabolic pathways using the KEGG metabolic framework as shown in Figure 6A. In fecal pathway analysis, comparison 1 indicated four significantly upregulated metabolic pathways including nitrogen metabolism, the phospholipase D signaling pathway, the Foxo signaling pathway, and D-alanine metabolism. Meanwhile, comparison 2 showed that insulin resistance, type II diabetes mellitus, the pentose phosphate pathway, the AMPK signaling pathway, arginine and proline metabolism, and insulin secretion were upregulated, while only the melanogenesis pathway was downregulated in the T2D + OMV group. In gut pathway analysis, comparison 3 indicated that D-arginine and D-ornithine metabolism and the TCA cycle were upregulated, while thyroid hormone synthesis was downregulated in obese mice with OMV intervention. In comparison 4, insulin secretion and the regulation of lipolysis were upregulated, whilst the AMPK signaling pathway was downregulated in the T2D + OMV group. For the liver pathway analysis, comparison 5 indicated six significantly upregulated metabolic pathways including Fc gamma R-mediated phagocytosis, the Fc epsilon RI signaling pathway, arachidonic acid metabolism, the regulation of lipolysis, the GnRH signaling pathway, and aldosterone synthesis. Moreover, comparison 6 identified eight significantly upregulated metabolic pathways including oxidative phosphorylation, the glucagon signaling pathway, propanoate metabolism, pyruvate metabolism, the TCA cycle, arginine and proline metabolism, the cAMP signaling pathway, and the mTOR signaling pathway.

Then we annotated the metabolic pathways most significantly affected in comparisons 1–6, and the results showed that these metabolic pathways involved the 21 most relevant metabolites which included l-glutamate, 4-aminobutanoate, d-alanine, quinolinate, glycine, glutathione disulfide, 5-oxoproline, pyruvate, l-proline, sarcosine, creatinine, l-tyrosine, 2-oxoglutarate, succinate, fumarate, citrate, cis-aconitate, d-alanyl-d-alanine, arachidonate, (r)-3-hydroxybutanoate and l-leucine (Figures 6B–G).

### 3.8. Gut microbiota data

#### 3.8.1. Gut microbiota in the five groups

**Figure 7:**
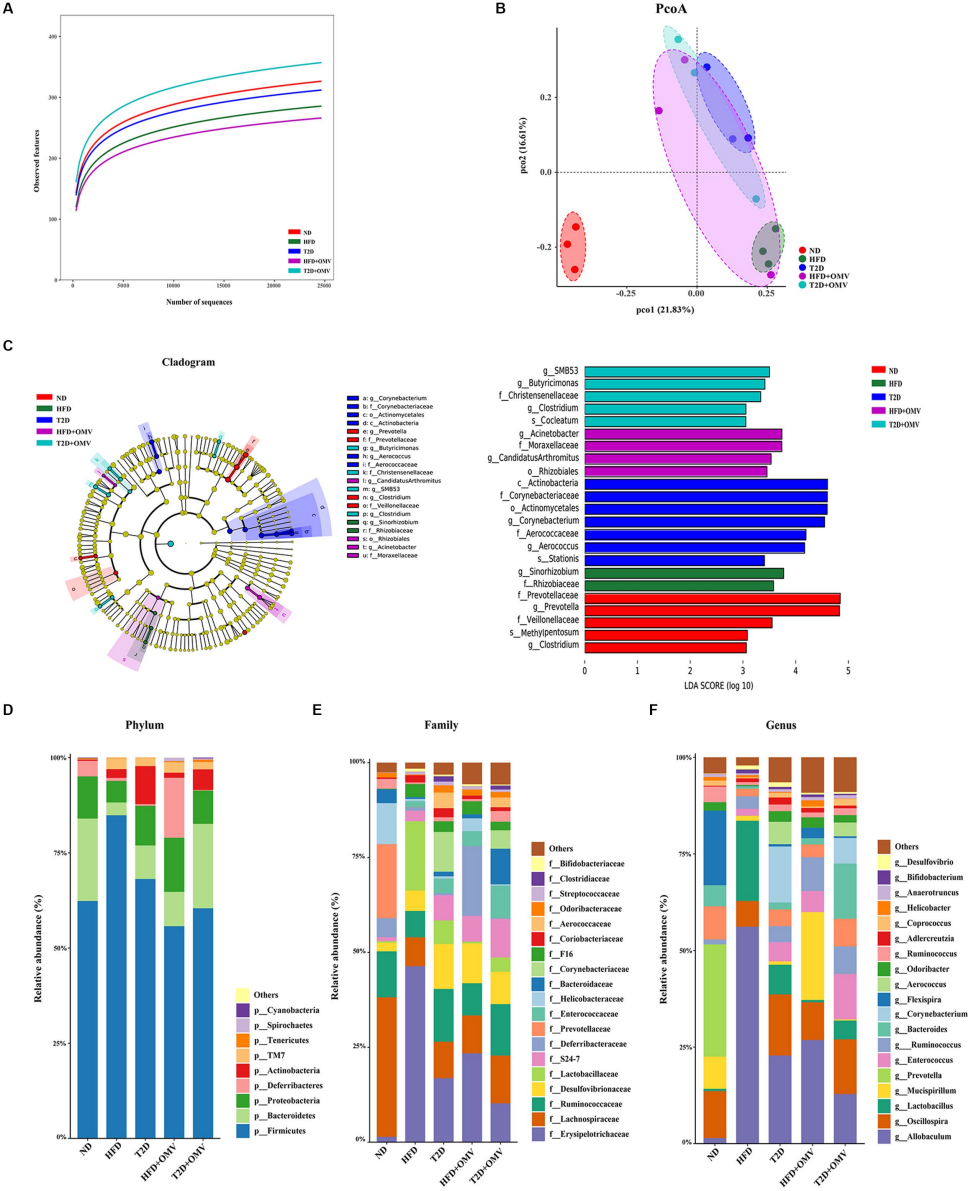
Gut microiota. The intestinal microbiota was different in mice in the different groups: normal diet (ND, red), high-fat diet (HFD, green), type 2 diabetes (T2D, blue), high-fat diet + OMVs (HFD + OMV, purple), and type 2 diabetes + OMVs (T2D + OMV, cyan). **(A)** The alpha diversities of gut microbes in the five groups. **(B)** PCoA plots based on bray metrics. **(C)** In the LEfSe cladogram, the inner to outer radiating circles represent the taxonomic level from phylum to species, and the diameter of the circles is proportional to the relative abundance. Species with no significant differences between groups are colored yellow, and species with significant differences are colored according to the group. Only taxa with *p* < 0.05 and LDA score (log_10_) are shown. The relative abundance of species is shown at the **(D)** phylum, **(E)** family and **(F)** genus levels across the five groups. ND, normal diet; HFD, high-fat diet; T2D (type 2 diabetes), high-fat diet + STZ; HFD + OMV, high-fat diet + OMVs; T2D + OMV, high-fat diet + STZ + OMVs.

Sequencing of the V3-V4 region of the 16S rRNA gene was performed on fecal samples. The alpha diversities of the gut microbiota analyzed using rarefaction curves showed marked differences of microbial species diversity between the five groups (Figure 7A). The gut microbiota diversity of the HFD group and T2D group was lower than that for the ND group. Interestingly, the alpha diversity was lower in the HFD group after OMV treatment, while the alpha diversity was higher in the T2D group after OMV intervention, indicating divergent modulations of the intestinal flora by OMVs. The principal coordinate analysis (PCoA) revealed that the normal diet group were separated from the other four groups (Figure 7B). The significant differences in bacteria among the five groups were shown by LEfSe analysis (Figure 7C). The differentially abundant bacteria were *Sinorhizobium* and *Rhizobiaceae* in the HFD group, but were *Rhizobiales, Acinetobacter and Moraxellaceae* in the HFD + OMV group. Then the differentially abundant bacteria were *Corynebacterium, Corynebacteriaceae, Actinomycetales, Actinobacteria, Aerococcus and Aerococcaceae in the T2D group*, but were *Butyricimonas, Christensenellaceae, SMB53 and Clostridium* in the T2D + OMV group. Finally, *Prevotella*, *Prevotellaceae*, *Clostridium* and *Veillonellaceae* were the markers in the ND group. These findings showed that the OMV treatments were capable of influencing the compositions of intestinal flora in the HFD group and the T2D group.

#### 3.8.2. Gut microbial changes at phylum, family and genus levels

The bacterial composition of the gut contents of the mouse groups was determined at the phylum, family and genus levels by 16S rRNA sequencing (Figures 7D–F).

At the phylum level, *Firmicutes* and *Bacteroidetes* accounted for more than 75% of the bacterial abundance for groups without OMV treatment (Figure 7D). *Firmicutes* accounted for 62.33%, 68.12%, and 84.84% in the ND, T2D, and HFD groups, respectively. *Bacteroidetes* accounted for 21.65%, 8.79%, and 3.36% in the ND, T2D, and HFD groups, respectively. Consistently, analysis of the 16S rRNA data using ANCOM showed that *Firmicutes* and *Bacteroidetes* accounted for the most number of differentially abundant OTUs at the phylum level when compared among HFD vs. ND, T2D vs. ND, HFD + OMV vs. HFD, and T2D + OMV vs. T2D (Table 1). Of note, the *Firmicutes*/*Bacteroidetes* ratios increased in the order of the ND, T2D, and HFD groups. After OMV treatment, the proportion of *Firmicutes* decreased in the HFD + OMV group (55.73%) and the T2D + OMV (60.42%) group. Meanwhile, the proportion of *Bacteroidetes* increased after OMV treatment in the HFD + OMV group (9.02%) and the T2D + OMV group (22.16%). The *Firmicutes*/*Bacteroidetes* ratios for the HFD + OMV group and the T2D + OMV group were similar to the ND group. Therefore, different diets and OMV treatments could alter the composition of the major intestinal microbiota.

**Table 1:** Differentially abundant OTUs at the phylum level were identified by ANCOM when comparing mice faeces collected from ND, HFD, T2D, HFD + OMV, and T2D + OMV groups.

HFD vs. ND		T2D vs. ND		HFD + OMV vs. HFD		T2D + OMV vs. T2D	
Number of OTUs considered = 549		Number of OTUs considered = 564		Number of OTUs considered = 425		Number of OTUs considered = 497	
Phylum	Sig	Phylum	Sig	Phylum	Sig	Phylum	Sig
Actinobacteria	19	Actinobacteria	14	Actinobacteria	7	Actinobacteria	4
Bacteroidetes	34	Bacteroidetes	48	Bacteroidetes	10	Bacteroidetes	8
Cyanobacteria	1	Cyanobacteria	1	Deferribacteres	1	Cyanobacteria	1
Firmicutes	161	Firmicutes	171	Firmicutes	37	Firmicutes	33
Proteobacteria	24	Proteobacteria	35	Proteobacteria	6	Proteobacteria	8
TM7	5	TM7	5	Tenericutes	1	Tenericutes	1
Tenericutes	4	Tenericutes	3				
Total	248	Total	277	Total	62	Total	55

The sig represents significantly different OTUs.

At the family level (Figure 7E), *Erysipelotrichaceae*, *Lachnospiraceae*, *Ruminococcaceae*, *Desulfovibrionaceae*, and *Lactobacillaceae* were the most abundant bacterial taxa. Compared with the non-OMV treatment groups (HFD = 46.34%, T2D = 16.86%), *Erysipelotrichaceae* was reduced in the OMV treatment groups (HFD + OMV = 23.41%, T2D + OMV = 10.24%). *Lachnospiraceae* in the OMV treatment groups (HFD + OMV = 9.99%, T2D + OMV = 12.58%) was more abundant than in the groups without OMV treatment (HFD = 7.64%, T2D = 9.59%).

At the genus level (Figure 7F), *Allobaculum*, *Oscillospira*, *Lactobacillus*, *Mucispirillum*, and *Prevotella* were the most abundant genera., *Allobaculum* was reduced in the OMV treatment groups compared with the goups without OMV treatment (HFD = 56.14% vs. HFD + OMV = 26.81%, T2D = 22.80% vs. T2D + OMV = 12.84%). Likewise, *Lactobacillus* had reduced abundance in all OMV treatment groups compared with the goups without OMV treatment (HFD = 20.78% vs. HFD + OMV = 0.60%, T2D = 7.69% vs. T2D + OMV = 4.87%).

### 3.9. Correlation between stool metabolites and gut microbiota

**Figure 8:**
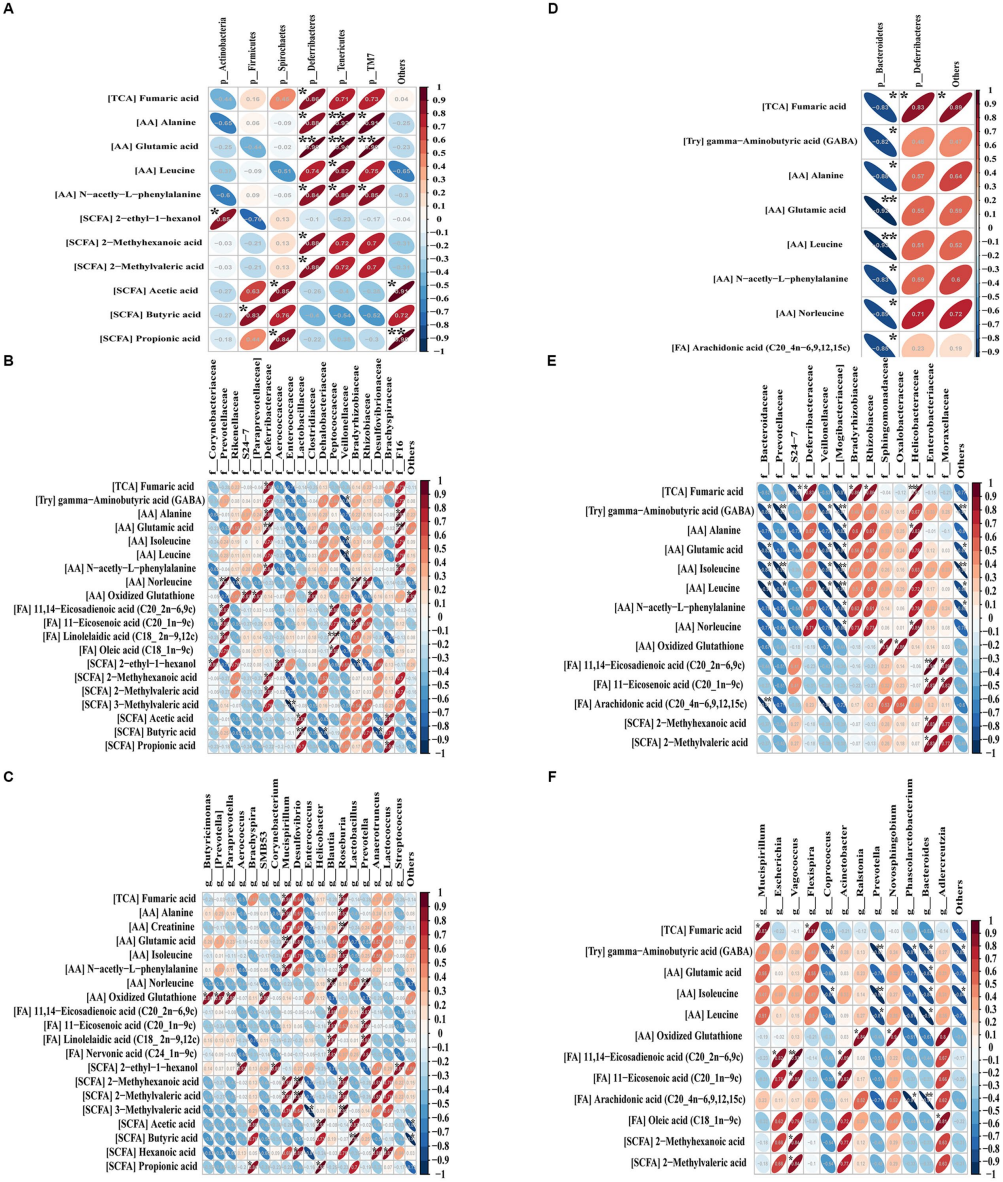
Pearson’s correlations between the significant metabolites and dominant gut flora at the phylum **(A)**, family **(B)** and genus **(C)** levels before or after **(D–F)** OMV treatment. Blue diagonal down ellipses and red diagonal up ellipses represent negative and positive relationships between metabolites and microbiota, respectively. ^*^p < 0.05, ^**^p < 0.01, and ^***^p < 0.001.

In order to investigate potential associations between intestinal flora and stool metabolites, Pearson correlation coefficients were determined between shortlisted metabolites (Figure 5E) and dominant gut flora at the phylum, family, and genus levels (Figures 7D–F). Charts of intestinal flora-host metabolites interactions before or after the EcN-OMVs treatment are presented in Figure 8.

At the phylum level before OMVs treatment (Figure 8A), a total of six phyla were positively correlated with 11 differentially abundant metabolites, including *Actinobacteria* with 2-ethyl-1-hexanol, *Firmicutes* with butyric acid, *Spirochaete*s with acetic acid and propionic acid, *Deferribacteres* with six shortlisted metabolites (fumaric acid, alanine, glutamic acid, n-acetyl-L-phenylalanine, 2-methyl hexanoic acid and 2-methyl valeric acid), *Tenericutes* with four shortlisted metabolites (alanine, glutamic acid, leucine, and n-acetyl-l-phenylalanine), and *TM7* with three shortlisted metabolites (alanine, glutamic acid, and n-acetyl-l-phenylalanine). After OMV treatment (Figure 8D), *Bacteroidetes* was significantly negatively correlated with eight differential metabolites (fumaric acid, gamma−aminobutyric acid, alanine, glutamic acid, leucine, n-acetyl-l-phenylalanine, norleucine, and arachidonic acid), while *Deferribacteres* was solely positively correlated with fumaric acid.

At the family level before OMV treatment (Figure 8B), a total of 18 families had significant correlations with 11 differential metabolites. Bacteria such as *Prevotellaceae*, *Deferribacteraceae*, *Peptococcaceae*, *Bradyrhizobiaceae* and *F16* showed positive relationships with nine metabolites (fumaric acid, alanine, glutamic acid, n-acetyl-L-phenylalanine, norleucine, oxidized glutathione, 11,14-eicosadienoic acid, 11-eicosenoic acid, and linolelaidic acid). *Veillonellaceae* showed negative relationships with three differential metabolites (gamma-aminobutyric acid, isoleucine, and leucine). After OMV treatment (Figure 8E), 14 families were correlated with 14 significant metabolites. Bacteria including *Deferribacteraceae*, *Bradyrhizobiaceae*, *Rhizobiaceae*, *Sphingomonadaceae*, *Oxalobacteraceae*, *Helicobacteraceae*, *Enterobacteriaceae,* and *Moraxellaceae* showed positive relationships with eight differential metabolites (fumaric acid, alanine, norleucine, oxidized glutathione, 11,14-eicosadienoic acid, 11-eicosenoic acid, 2-methyhexanoic acid, and 2-methylvaleric acid). *Bacteroidaceae*, *Prevotellaceae*, *S24-7*, *Veillonellaceae,* and *Mogibacteriaceae* showed negative relationships with eight characteristic metabolites (fumaric acid, gamma-aminobutyric acid, alanine, glutamic acid, isoleucine, leucine, n-acetyl-L-phenylalanine, and norleucine).

At the genus level before OMV treatment (Figure 8C), a total of 18 genera were correlated with 20 metabolites. Bacteria like *Mucispirillum*, *Desulfovibrio*, *Blautia*, *Roseburia,* and *Prevotella* showed positive relationships with 11 characteristic metabolites (fumaric acid, alanine, creatinine, glutamic acid, isoleucine, n-acetyl-L-phenylalanine, norleucine, 11,14-eicosadienoic acid, 11-eicosenoic acid, linolelaidic acid, and nervonic acid). *Enterococcus* showed a negative correlation with 3-methylvaleric acid. After OMV treatment (Figure 8F), a total of 13 genera were correlated with 12 differential metabolites. Bacteria including *Vagococcus* and *Acinetobacter* showed positive correlations with two significant metabolites (11,14-eicosadienoic acid and 11-eicosenoic acid). *Coprococcus*, *Prevotella*, *Phascolarctobacterium,* and *Bacteroides* showed negative correlations with five differential metabolites (gamma-aminobutyric acid, glutamic acid, isoleucine, leucine, and arachidonic acid).

## 4. Discussion

EcN is a probiotic that improves microbiota balance and gastrointestinal homeostasis (Rozanska et al., 2014). OMVs are constitutively produced by Gram-negative bacteria and have an important role in bacteria-host interactions. However, little is known about the influence of probiotic OMVs on host physiology. In this study, we carried out a comparative analysis of the effects of EcN-OMVs on HFD mice and T2D mice to understand the mechanisms underlying effects on obesity and type 2 diabetes. Our findings indicated that administering EcN-OMVs could reduce body weight and blood glucose concentration, and increase plasma insulin levels. Through 16S rRNA gene sequencing analysis, we found that EcN-OMV treatments could modify the ratio of *Firmicutes*/*Bacteroidetes* in the intestine and modulate the relative abundance by increasing beneficial microbiota (*Lachnospiraceae* and *Oscillospira*) and inhibiting the growth of pathobiont bacteria (*Erysipelotrichaceae*). The fecal metabolome showed that EcN-OMVs might regulate SCFA concentrations in the intestinal by reducing the abundance of SCFA-production bacteria. Gut metabolome analysis suggested that EcN-OMVs might influence the intestinal ornithine and fumaric acid levels to mediate the gut ornithine cycle. Liver metabolome analysis revealed that EcN-OMVs might reduce hepatic steatosis in HFD mice by reducing ω-6 unsaturated fatty acid metabolism in the liver. Thus, EcN-OMVs seem to ameliorate the pathophysiologiy of obesity and diabetes by modulating gut-hepatic homeostasis.

### 4.1. The beneficial effects of EcN-OMVs on obese and T2D mice

Recent reports have confirmed the direct association between gut microbiota-derived OMVs and metabolic diseases, including obesity and diabetes (Nah et al., 2019; Diez-Sainz et al., 2022). Consistent with those studies, we found that oral administration of OMVs led to a significant reduction in body weight and hepatic lipid droplets in both HFD and T2D groups (Figures 3B, 4A). Although no study has investigated the effects of EcN-OMVs on obesity and diabetes, several studies have shown the beneficial outcomes of administering other Gram-negative bacteria on metabolic disorders. Chelakkot et al. (2018) showed that oral administration of *Akkermansia muciniphila* (*A. muciniphila*) OMVs to HFD-fed mice decreased gut barrier permeability, reduced body weight gain, and improved glucose tolerance. Ashrafian et al. (2019) demonstrated that *A. muciniphila* OMVs reduced body weight, lowered adiposity, and ameliorated intestinal inflammation in HFD-induced obese mice. In our study, mice fed with a HFD were prone to the effects of OMVs, including profoundly reduced blood glucose concentrations and elevated plasma insulin levels, with a similar trend in the T2D group. The less significant effect in the T2D group may be due to the fact that the HFD group was only perturbed by the dietary intervention, while the T2D group was administrated STZ in addition, which causes permanent destruction of mouse islet β cells (Szkudelski, 2001; Gheibi et al., 2017). Thus, the EcN-OMV treatment is effective in ameliorating the adverse outcomes caused by a high-fat diet.

### 4.2. Effects of EcN-OMVs on the gut flora of obese and T2D mice

The gut microbiota plays a crucial role in the modulation of host physiological processes the alterations of which have been strongly associated with the onset and progression of obesity and diabetes (Salgaco et al., 2019; Singer-Englar et al., 2019). Growing evidence suggests that gut microbiota-derived OMVs could be important mediators in gut microbiota-intestinal homeostasis and ultimately influence the pathogenesis of metabolic diseases (Diez-Sainz et al., 2022). Hence, we performed 16S rRNA sequencing of stool samples in order to determine variations in the gut microbiota canused by EcN-OMV administration in obese and diabetic mice.

Our results demonstrated that taxonomic spectra were distinctly different among the five experimental groups from the genus to phylum level (Figures 7D–F), indicating strong perturbations of the intestinal flora and marked regulation by EcN-OMVs. Specifically, at the phylum level before EcN-OMVs treatment with a normal diet, *Bacteroidetes* (B) and *Firmicutes* (F) were the dominant microbes, and the HFD and T2D groups had an increased F/B ratio, due to more *Firmicutes* and less *Bacteroidetes*, which was consistent with previous reports (Vemuri et al., 2018). Coincidentally, this increased F/B ratio has been reported to possibly lead to excessive low-grade inflammation (Wen and Duffy, 2017). Moreover, we found that *Firmicutes* and *Spirochaetes* had positive relationships with inflammatory-related short-chain fatty acids (SCFAs) like acetic acid, butyric acid, and propionic acid (Figure 8A). This result indicated that the HFD reshaped the gut flora such that it produced some proinflammatory SCFAs. After EcN-OMV treatment the F/B ratio at the phylum level decreased in both the HFD and T2D groups. In addition, *Bacteroidetes* abundance showed a negative correlation with metabolites such as arachidonic acid and leucine (Figure 8B). Previous studies have shown that arachidonic acid and its downstream oxylipins play a crucial role in the pathobiology of diabetes mellitus (Das, 2018). In addition, leucine concentrations have been reported to be altered between obese and low BMI humans and contribute to insulin resistance (Newgard et al., 2009). Thus, we postulate that EcN-OMVs might influence intestinal flora composition and subsequently generated anti-inflammatory metabolites that reduce insulin resistance and obesity.

Our study found that two significant bacterial families *Erysipelotrichaceae* and *Lachnospiraceae* which belong to the *Firmicutes* phylum play an important role in ameliorating obesity and diabetes. Firstly, the proportion of *Erysipelotrichaceae* in the HFD and T2D groups was higher than in the ND group prior to EcN-OMV treatment, while it was reduced in both of the high fat-diet groups (HFD + OMV&T2D + OMV) after OMV treatment. Reports documenting a potential role for *Erysipelotrichaceae* in host physiology and disease are increasing. For example, Chen et al. (2018) have shown that an HFD may increase the relative abundance of *Erysipelotrichaceae* in animals and individuals. Spencer et al. (2011) demonstrated that the abundance of *Erysipelotrichaceae* was positively associated with fatty liver in humans. In addition, Zschaler et al. (2014) and Palm et al. (2014) have found that species within the *Erysipelotrichaceae* phylum may have diverse immunogenicity profiles or respond differently to inflammation within the gut. We found that when we instituted a high-fat diet for the HFD and T2D groups, the proportion of *Lachnospiraceae* decreased significantly in comparison with the ND group. A decrease in *Lachnospiraceae* abundance is likely to have negative health implications resulting from the loss of the numerous beneficial functions performed by members of this family. For example, *Lachnospiraceae* can contribute to the microbiota’s resistance to colonization by drug-resistant pathogens through the conversion of primary to secondary bile acids, and production of the SCFAs acetate and butyrate (Buffie et al., 2015). Interestingly, the level of *Lachnospiraceae* increased in both the HFD + OMV and T2D + OMV groups after OMV administration. Therefore, the positive regulation of intestinal *Erysipelotrichaceae* and *Lachnospiraceae* levels may be an important mechanism by which EcN-OMVs ameliorate obesity and diabetes.

### 4.3. Effects of EcN-OMVs on the fecal metabolome of obese and T2D mice

Numerous studies have confirmed that intestinal bacteria-derived SCFA metabolites play an important role in obesity and diabetes. Changes in the microbiota and SCFA profile are profoundly associated with host metabolism. For instance, SCFAs are involved in various physiological functions, including providing energy to intestinal cells, maintaining intestinal mucosal barrier and immune function, and regulating blood sugar and insulin levels (van der Hee and Wells, 2021). Our study found that after instituting a high-fat diet, the abundance of *Firmicutes* in the HFD and T2D mice increased and the abundance of *Bacteroides* decreased. Joseph et al. (2019) have shown that *Firmicutes* and *Bacteroides* are involved in microbial dysbiosis and the development of obesity, and a higher concentration of SCFAs in the feces of overweight children than in healthy children. Gomes et al. (2018) and Iatcu et al. (2021) demonstrated that an imbalance of the intestinal microbiome and a decrease in the amount of SCFA often occurs in patients with obesity or T2D. Qin et al. (2010) suggested that the abundance of SCFA-producing bacteria was decreased in the gut microbiota of T2D patients.

Furthermore, intestinal SCFA levels can be modulated by probiotics, which could help restore intestinal homeostasis. We found that after OMV intervention the abundance of *Bacteroides*, *Lachnospiraceae*, and *Oscillospira* increased in both HFD and T2D groups. Ashrafian et al. found that the abundance of *Bacteroides* could affect intestinal SCFAs when exposed to *Akkermansia muciniphila* extracellular vesicles. The same authors also reported that the level of *Bacteroides* was negatively correlated with colonic inflammation and proinflammatory cytokines in obese mice (Ashrafian et al., 2021). Byndloss et al. (2017) showed that *Lachnospiraceae* could influence acetate and butyrate production. Yang et al. (2021) demonstrated that *Oscillospira* was negatively associated with obesity and obesity-related chronic inflammatory and metabolic diseases. Meanwhile, Konikoff and Gophna (2016) have shown that *Oscillospira* is also likely to be a genus capable of producing SCFAs dominated by butyrate. In summary, OMVs may alleviate intestinal flora disturbance in obese and diabetic mice caused by a high-fat diet and increase the abundance of SCFA-producing bacteria.

### 4.4. Effects of EcN-OMVs on the gut metabolome of obese and T2D mice

Among the significant changes (*p* < 0.05, FC > 1.5) in intestinal metabolite concentrations, only ornithine was higher in the HFD + OMV group compared to non-OMV treatment (Figure 5F). Ornithine is a non-essential amino acid synthesized by the enzymatic action of arginase on arginine as part of the urea cycle (Sivashanmugam et al., 2017). Ornithine in the intestine comes from dietary intake (protein-rich food), and endogenous synthesis occurs primarily in the gut microbiota through ornithine synthetases. Notably, Bergen and Wu (2009) suggested that microorganisms play an important role in the intestinal nitrogen cycle, including deaminating ornithine/arginine, hydrolyzing luminal urea, and reabsorbing ammonia. Our study found that arginine and ornithine metabolism was upregulated in gut tissue after OMV administration, the F/B ratio was decreased and the abundance of *Bacteroides* increased significantly. Yoshida et al. (2021) demonstrated that an increased abundance of *Bacteroides* has been negatively associated with obesity. Wexler reported a significant correlation between nitrogen utilization efficiency with the presence of *Bacteroides*. For example, *Bacteroides* could synthesize and degrade amino acids, using them as a nitrogen source to synthesize microbial proteins. It could also undertake several nitrogen metabolic pathways, such as the urea cycle and nitrate metabolism, to regulate the utilization and excretion of nitrogen in the gut (Wexler, 2007). Furthermore, Qi et al. (2019) demonstrated that the modulation of the gut nitrogen cycle through arginine and ornithine could benefit gut mucosal barrier function. Cynober (1994) found that continuous feeding of ornithine to hungry rats can lead to a significantly higher crypt height in the jejunum and ileum and a higher total villous height in the ileum. Ornithine has been shown to maintain the integrity and normal morphology of the intestinal barrier by regulating the secretion of the mucus layer and the proliferation of intestinal epithelial cells. Thus, OMVs might influence the intestinal ornithine level by affecting the gut nitrogen cycle through modulation of microbial metabolism. However, the exact mechanism by which OMVs modulate gut nitrogen metabolism requires further investigation.

In our study, the fumaric acid concentration was significantly higher in the T2D + OMV group than in the T2D group and both the insulin secretion pathway and the lipolysis pathway were upregulated after OMV administration (Figures 5F, 6A). STZ is well known to cause pancreatic β-cell damage and can also inhibit the activity of enzymes that participate in the TCA cycle (Lenzen, 2008). Rojas et al. (2019) found that mice at 12 weeks post-STZ treatment showed an early TCA cycle impairment, and the fumaric acid level was significantly reduced. Some studies have demonstrated that bacterial OMVs contains proteins and enzymes related to the TCA cycle and oxidative phosphorylation process, such as NADH dehydrogenase and ATP synthase (Lee et al., 2007; Aguilera et al., 2014). Furthermore, Qiao et al. (2016) revealed that TCA cycle intermediates (fumaric acid, malic acid, citric acid, and succinic acid) in adipocytes exhibited oscillatory changes over time in response to insulin. Together, these results suggest that EcN-OMVs might alleviate TCA cycle damage caused by STZ by increasing fumaric acid concentrations.

### 4.5. Effects of EcN-OMVs on the liver metabolome of obese and T2D mice

The liver is a critical hub for numerous physiological processes. These include regulating glucose metabolism and lipid metabolism, and participating in inflammation and immune regulation. In this study, the mice’s hepatic metabolome profiles were analyzed prior to, and post, OMV treatment. We found that the concentrations of four omega-6 (ω-6) unsaturated fatty acids (11, 14-eicosadienoic acid, 13, 16 − docosadienoic acid, dihomo-γ-linoleic acid, and arachidonic acid) were significantly increased in the HFD + OMV group. ω-6 fatty acids are a family of essential fatty acids that act as precursors for inflammatory metabolites. In particular, γ-linoleate is converted into dihomo-γ-linoleic acid and then desaturated to arachidonic acid, which subsequently serves as the precursor for the biosynthesis of inflammatory eicosanoids (e.g., prostaglandins) by cyclooxygenase-2 (COX-2). Several studies have demonstrated that SCFAs could inhibit the activity of the COX-2 enzyme (Liu et al., 2016; Kurata et al., 2019). Therefore, we believe that EcN-OMVs might suppress the activity of oxygenases involved in ω-6 unsaturated fatty acid metabolism by increasing SCFA concentrations in the liver, leading to the accumulation of ω-6 unsaturated fatty acids in the liver and reducing inflammation. However, the specific mechanism remains unknown.

**Figure 9:**
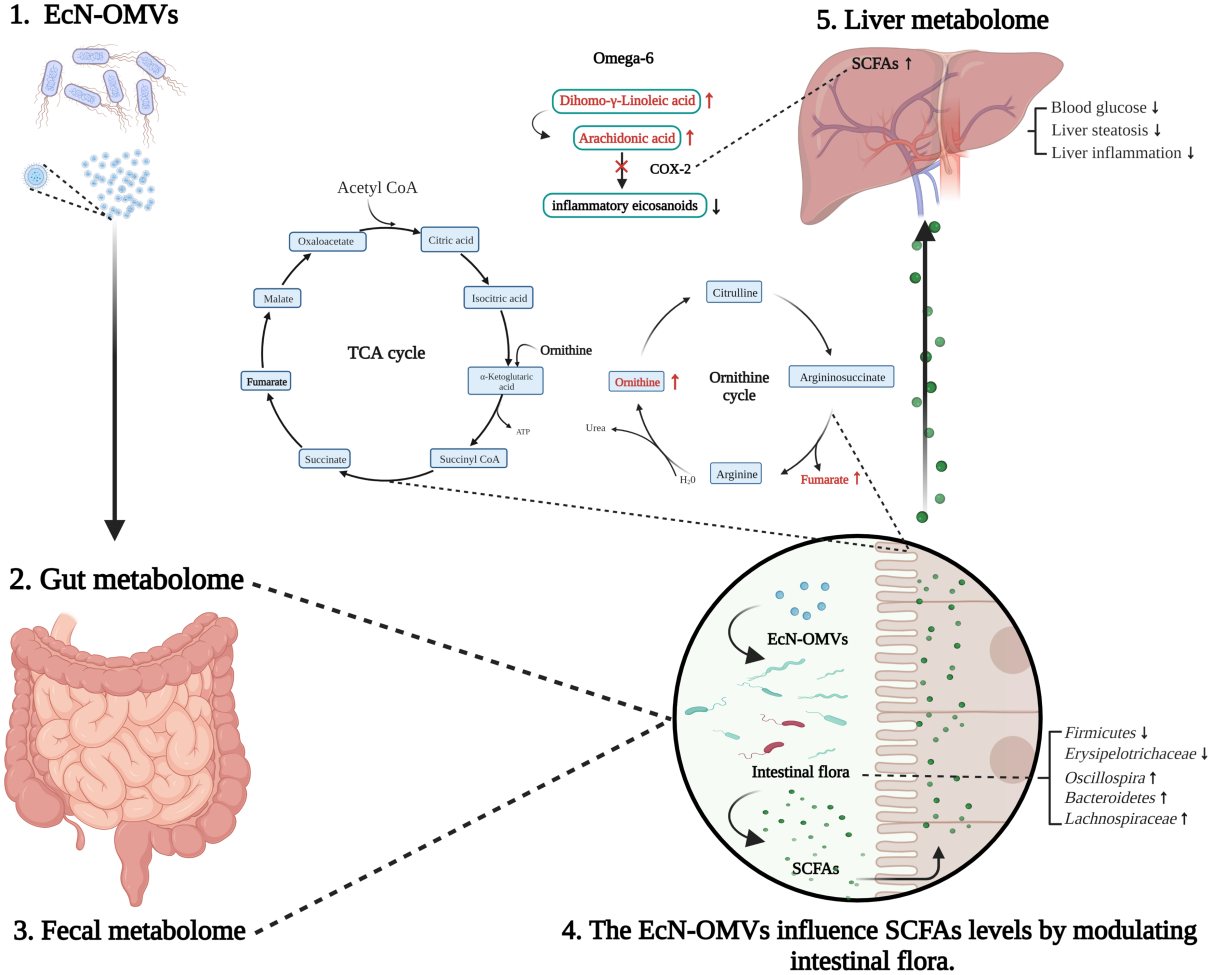
Summary of EcN-OMV regulation of the intestinal flora and gut-liver metabolism in obese and diabetic mice. Firstly, EcN-OMV administration modifies the ratio of Firmicutes/Bacteroidetes in the gut. This leads to the elevation of short-chain fatty acid (SCFA)-producing flora and increasing the concentrations of SCFAs in the intestine. Furthermore, intestinal ornithine cycle intermediates such as ornithine and fumaric acid are altered in response to EcN-OMV administration. Then, SCFAs are transported from the intestine to the liver through the hepatic portal vein. Finally, higher hepatic SCFA concentrations may suppress COX2 and subsequently downregulate the ω-6 unsaturated fatty acid metabolism. The upward arrow indicates a higher concentration/upregulation, and the downward arrow indicates a lower concentration/downregulation.

Furthermore, SCFAs seem to act as signaling molecules between the gut microbiota and their host. We found that the concentration of 2-methyl-hexanoic acid was significantly changed in both the fecal and hepatic metabolome. den Besten et al. (2015) suggested that SCFAs appear to regulate hepatic lipid and glucose homeostasis in an adenosine monophosphate-activated protein kinase-dependent manner involving peroxisome proliferator-activated receptor-g regulated effects on gluconeogenesis and lipogenesis. Moreover, Chambers et al. (2015) demonstrated that increased SCFA flux through the liver could reduce intrahepatic triglyceride concentrations, likely improving hepatic fat accumulation. Indeed, our results showed the number of liver adipocytes in obese mice significantly decreased, and the intestinal SCFA-producing flora increased after administering EcN-OMVs. Microbiota-derived SCFAs are absorbed in the intestine and transported through the hepatic portal system to modulate liver metabolism. Thus, EcN-OMVs might reduce hepatic steatosis in HFD mice by increasing the concentration of ω-6 unsaturated fatty acids and SCFAs in the liver, and SCFAs are key molecules connecting liver and intestinal metabolomes (Figure 9).

### 4.6. Limitations

Despite the promising results, several limitations of our research merit discussion. Firstly, EcN has been shown to directly modulate host glucose metabolism and improve postprandial glycemic response in mice (Chavkin et al., 2021). Thus comparisons between EcN and EcN-OMV administrations in obese and diabetic mice should be performed to evaluate their similar or different effects on host glucose metabolism. Secondly, we have isolated EcN-OMVs using the established method of differential ultracentrifugation. Nevertheless, it has been reported that the size-based bacterial OMV separation co-isolated protein contaminants, while size-based tangential flow filtration (TFF) followed by charge-based high-performance anion exchange chromatography (HPAEC) enhances purity (Piroli et al., 2023). Future work should implement orthogonal TTF with HPAEC to purify EcN-OMVs.

## 5. Conclusion

This is the first study of the effects of EcN-OMVs in obese and diabetic mice based on gut-liver axis metabolomics combined with analysis of the gut microbiome. Overall, our findings have demonstrated that EcN-OMVs can regulate intestinal and liver metabolism by affecting gut microbiota and SCFA concentrations. Thus, this study has laid the foundation for applying EcN-OMVs as a post-biotic agent and potential adjuvant treatment for obesity and diabetes.

## Data availability statement

The availability of 16S rRNA gene sequences in this study are deposited in the NCBl Sequence Read Archive database, accession number PRJNA971528.

## Ethics statement

The animal studies were approved by Institutional Animal Care and Use Committee (IACUC) of the Chongqing Medical University. The studies were conducted in accordance with the local legislation and institutional requirements. Written informed consent was obtained from the owners for the participation of their animals in this study.

## Author contributions

JS contributed to the sample and data collection, performed the statistical analysis, interpreted the results, and wrote the manuscript. DM contributed to interpreting the results and wrote the manuscript. SG, FL, XW, and XP contributed to the samples collection. RC commented on the experimental design and revised the manuscript. T-LH devised the original laboratory study, interpreted the results, supported the writing of the manuscript, directed the project, guarantor of this work and, as such, had full access to all the data in the study and takes responsibility for the integrity of the data and the accuracy of the data analysis. All authors contributed to the article and approved the submitted version.

## Funding

This study was supported by the Foundation of State Key Laboratory of Ultrasound in Medicine and Engineering (2023KFKTOO2), Chongqing Science & Technology Commission (cstc2021jcyj-msxmX0213), Chongqing Municipal Education Commission (KJZD-K202100407), and Senior Medical Talents Program of Chongqing for Young and Middle-aged (2022) 15, and the Kuanren Talents Program of the Second Affiliated Hospital of Chongqing Medical University.

## Conflict of interest

The authors declare that the research was conducted in the absence of any commercial or financial relationships that could be construed as a potential conflict of interest.

## Publisher’s note

All claims expressed in this article are solely those of the authors and do not necessarily represent those of their affiliated organizations, or those of the publisher, the editors and the reviewers. Any product that may be evaluated in this article, or claim that may be made by its manufacturer, is not guaranteed or endorsed by the publisher.
